# Adherence to the treatment of gestational syphilis: influence of psychosocial, socioeconomic, and clinical factors

**DOI:** 10.1016/j.jped.2026.101566

**Published:** 2026-06-05

**Authors:** Alessandra Andrade Fantinelli, Martina Alana Lodi, Cristiane Athanasio Kolbe, Ivana de Souza Karl, Thiago Wendt Viola

**Affiliations:** a Pontifícia Universidade Católica do Rio Grande do Sul (PUCRS), Escola de Medicina, Programa de Pós-Graduação em Pediatria e Saúde da Criança, Porto Alegre, RS, Brazil; b Grupo Hospitalar Conceição (GHC), Porto Alegre, RS, Brazil; c Hospital de Clínicas de Porto Alegre (HCPA), Porto Alegre, RS, Brazil

**Keywords:** Gestational syphilis, Adherence, Treatment, Prenatal care, Psychosocial

## Abstract

**Objective:**

To identify psychosocial, sociodemographic, and clinical factors associated with adherence to gestational syphilis treatment.

**Method:**

cross-sectional study with a sample of 325 pregnant women diagnosed with syphilis during pregnancy. The main outcome was complete adherence to the therapeutic regimen of three doses of penicillin, including the partner’s treatment. Data were collected through structured interviews and medical records. Statistical analysis included logistic regressions and co-occurrence network analysis to explore interrelationships between factors associated with treatment adherence.

**Results:**

Treatment adherence was observed in 43.6% of pregnant women and their partners. Newborns of mothers who adhered to treatment had significantly higher Apgar and Capurro scores. Positive factors associated with adherence included early diagnosis in the first trimester (odds ratio [OR]: 5.12, 95% confidence intervals [CI]: 2.8–9.7), adequate prenatal follow-up (OR: 4.37, CI: 2.5–7.7), planned pregnancy (OR: 2.36, CI: 1.3–4.1), employment (OR: 1.72, CI: 1.1–2.7), and income above the minimum wage (OR: 1.71, CI: 1.1–2.6). Negative factors included a history of substance misuse during life and pregnancy (OR: 0.34, CI: 0.1–0.7), childhood maltreatment (OR: 0.53, CI: 0.3–0.8), recurrent unprotected sex (OR: 0.61, CI: 0.3–0.9), marital conflicts (OR: 0.47, CI: 0.3–0.7), witnessed violence (OR: 0.51, CI: 0.3–0.8), and prior police detention (OR: 0.29, CI: 0.1–0.5). Network analysis revealed three clusters related to these factors: one associated with violence, another with substance use, and another involving socioeconomic factors and prenatal care access.

**Conclusion:**

Adverse maternal psychosocial factors, such as violence, maltreatment, and substance use, hamper treatment adherence and may contribute to worse neonatal outcomes.

## Introduction

Between 2005 and June 2024, 713,167 cases of syphilis in pregnant women were reported in Brazil’s National Notifiable Diseases Information System. In 2023, Brazil recorded a rate of 34 cases per 1000 live births, representing a 3.3% increase compared to the previous year [1]. According to the World Health Organization, Brazil has a substantial burden of syphilis, comparable to that observed in other middle-income countries with similar epidemiological profiles [2]. In this context, reducing incidence will also depend on timely testing and treatment and sustained adherence to the recommended regimen [3].

Treatment for syphilis is primarily performed through the administration of benzathine penicillin, an effective antibiotic therapy that eradicates *Treponema pallidum* [4]. Furthermore, it is essential that the sexual partner is also evaluated and treated, even in the absence of symptoms, to prevent reinfection. If the partner does not receive adequate treatment, there is a risk of reinfecting the treated individual, perpetuating the transmission cycle, and increasing disease incidence within the community [5].

Several sociodemographic and clinical factors may contribute to difficulties in adhering to syphilis treatment, for example: lack of access to health services offering treatment, individuals who are very underweight with little muscle tissue for medication administration, distance to health facilities, financial difficulties for transportation, and unawareness of the availability of free prevention, diagnosis, and treatment supplies in public health units [6,7]. Beyond these, other psychosocial vulnerabilities may also be associated, such as limited education, substance use, legal issues, exposure to violence, little social or family support, and late diagnosis [8,9].

Few studies have evaluated the risk and protective factors that may influence treatment adherence within the Brazilian Unified Health System (SUS) [10]. Thus, the present study aims to identify various psychosocial, clinical, and sociodemographic factors associated with adherence to the treatment of syphilis during pregnancy, seeking to understand which of them contribute positively or negatively.

## Methods

This study followed the guidelines of the *Strengthening the Reporting of Observational Studies in Epidemiology* (STROBE) [11]. It was an observational, cross-sectional study, conducted in a public medium- and high-complexity hospital located in Porto Alegre, Rio Grande do Sul, Brazil, which belongs to the *Grupo Hospitalar Conceição* (GHC). All services are funded by the SUS.

### Sample

The study included 325 women aged 18 to 45 years with syphilis during pregnancy, confirmed by a reactive VDRL test. During hospitalization, a treponemal test (FTA-Abs or MHATP) was also requested regardless of titer, as part of the hospital's standard procedure. Women with a previous diagnosis of syphilis were eligible, irrespective of prior treatment, provided they had reactive VDRL and treponemal tests in the current pregnancy, confirming an active serological indication for treatment. Women whose partners had not completed treatment were also included. Exclusion criteria were acute confusion, cognitive impairment preventing the interview, or refusal to participate. Data were collected from May 2023 to October 2024. The study was approved by the institution’s Research Ethics Committee (No. 68,043,623.0.0000.5336; approval date: August 31, 2023), and all participants provided written informed consent.

The sample size estimation was based on previously published data on the prevalence of illicit drug use among patients diagnosed with syphilis (approximately 25%). Considering previous findings showing that around 52% of patients complete treatment, it was estimated that, to detect an odds ratio of 2.5 between illicit drug use and non-adherence to treatment, a minimum of 273 patients would be required to ensure a statistical power of 90% and a significance level of 5%.

### Data collection

Participants were invited to join the study during hospitalization for delivery, specifically between 24 and 48 h after birth. Refusal to participate after the initial invitation was low, accounting for <5% of approached women. Refused participants were not included in the analysis. At that point, trained members of the Mother-Baby Care Line Study Group at GHC approached eligible women, explained the study objectives, and obtained written informed consent before any data collection began. Interviews were conducted in a private setting within the maternity ward, ensuring confidentiality and minimizing social desirability bias in responses to sensitive questions (e.g., substance use, violence, childhood trauma). Data collection was conducted by members of the Mother-Baby Care Line Study Group at GHC, who underwent standardized training prior to the study to ensure consistent application of the interview protocol and instruments. Clinical data related to the prenatal period were obtained from the prenatal booklet and maternal medical records, complementing the structured interview. Perinatal and neonatal data were also extracted from medical records. The interview protocol combined two validated instruments in Brazilian Portuguese: the Addiction Severity Index, Sixth Edition (ASI-6) [12], and the Childhood Trauma Questionnaire (CTQ) [13].

The ASI-6, although originally developed for individuals with substance-related problems, assesses multiple psychosocial domains relevant to this study. In addition to substance use patterns, including drug, tobacco, and alcohol consumption, it evaluates employment and income, legal problems, family and social functioning, psychological symptoms, psychiatric treatment history, housing conditions, and lifetime exposure to violence. Its items also capture the number, extent, and duration of problems over the lifetime and in the 30 days prior to the interview.

The CTQ is a 28-item, five-point Likert scale that assesses childhood maltreatment across five domains: physical abuse, emotional abuse, sexual abuse, physical neglect, and emotional neglect. In this study, childhood maltreatment was considered present when the participant reported at least one response other than “never” in any item, and separate variables were created for each maltreatment type.

### Independent variables

Sociodemographic

• Maternal age (> 21 gt; 21 years): age in complete years at the time of care, categorized as below or above 21 years, which is used as a marker of full legal and social autonomy in the Brazilian context.
• Black ethnicity: self-declared identification as Black.
• Educational level: self-reported completion of secondary education.
• Living in the state capital: residence located in the state capital.
• Employment status: currently employed.
• Income above minimum wage: family income greater than one minimum wage, which reflects the official Brazilian poverty threshold used in national health and social policies.
• Stable relationship: being in a stable relationship.
• Receives government assistance: receiving government aid (e.g., *Bolsa Família*).
• Has more than one close friend: self-report of having more than one close friend, distinguishing individuals with some degree of social connectedness from those who are socially isolated.

Obstetric

• Previous parity (≥ 1 children): already has one or more children.
• Adequate prenatal care: more than six prenatal visits, following the minimum standard established by the Brazilian Ministry of Health's Prenatal Humanization Program (PHPN).
• Early diagnosis of syphilis: screening performed during the first trimester of pregnancy.
• Planned pregnancy: self-report of pregnancy as planned or intended.
• High-risk pregnancy: defined by the presence of comorbidities.

Psychological problems

• Lifetime psychiatric treatment: self-report of having undergone psychiatric treatment at any time.
• Lifetime diagnosis of depression/anxiety: self-report of a medical diagnosis of depression or anxiety.

Family, social, and legal variables

• Recent (last 30 days) conflict between intimate partners: recent arguments or fights with a partner.
• History of homelessness: self-report of having lived on the streets at any point in life.
• Recent (last 30 days) lack of social/family support: self-report of feeling unsupported socially or by family.
• History of police detention: detained by the police for at least one day.
• Child custody removal by child protection services: loss of custody at any point.

Trauma and violence

• Witnessed violence: witnessing severe violence.
• Victim of physical violence: experiencing physical violence.
• Victim of sexual violence: having suffered sexual violence.
• Childhood maltreatment (any type): exposure to abuse or neglect in childhood.
• Physical neglect in childhood: subcategory of maltreatment.
• Emotional neglect in childhood: subcategory of maltreatment.
• Physical abuse in childhood: subcategory of maltreatment.
• Emotional abuse in childhood: subcategory of maltreatment.
• Sexual abuse in childhood: subcategory of maltreatment.

Substance use

• Alcohol use disorder (lifetime): regular consumption (≥ 3 times per week for ≥ 1 year), which was the criterion derived directly from the ASI-6 instrument.
• Cannabis use disorder (lifetime): same criteria applied for cannabis.
• Tobacco use disorder (lifetime): same criteria applied for tobacco.
• Recent alcohol use (last 30 days): self-reported alcohol consumption within the last month.
• Recent cannabis use (last 30 days): same for cannabis.
• Recent tobacco use (last 30 days): same for tobacco.
• Substance use during pregnancy: report of cannabis or other drug use during pregnancy.
• Alcohol use during pregnancy: report of alcohol use during pregnancy.
• Tobacco use during pregnancy: report of tobacco use during pregnancy.
• History of treatment for alcohol/drug problems:previous specialized treatment for substance use.

Sexual behavior

• Early sexual debut: first sexual intercourse before 15 years old, which is widely used in the epidemiological literature as a marker of early sexual initiation and vulnerability to sexually transmitted infections, and is consistent with Brazilian legal definitions of sexual vulnerability under the Child and Adolescent Statute (ECA).
• Recent (last 30 days) unprotected sex: recent sexual intercourse without condom use.

### Outcome

The primary outcome was adequacy of treatment of the pregnant woman-partner dyad, defined as treatment of gestational syphilis with three doses of benzathine penicillin started at least 30 days before delivery, with a minimum interval of seven days between doses, according to the clinical stage of infection. Adherence was considered complete only when both the pregnant woman and her sexual partner received the appropriate regimen. Lack of partner treatment, even when the woman was adequately treated, was classified as non-adherence because of the risk of reinfection. For women without a current sexual partner, adherence was assessed based solely on the mother's treatment, as partner treatment was not applicable in these cases.

As a secondary outcome, groups with and without complete adherence were compared regarding neonatal outcomes, specifically Apgar score at the 1st minute and gestational age at birth estimated by the Capurro method.

### Biases

Self-reported data may be subject to recall and reporting bias, particularly for sensitive topics such as substance use, exposure to violence, and sexual behavior. Participants may have underreported stigmatized behaviors due to social desirability bias, fear of judgment, or concerns about legal consequences, which could lead to an underestimation of the prevalence of these exposures. Conversely, recall bias may affect the accuracy of retrospective reports, particularly for childhood maltreatment and lifetime exposure to violence. To mitigate these issues, trained interviewers applied validated and widely used instruments, the ASI-6 and the CTQ, which have been shown to improve disclosure of sensitive information. Additionally, the structured and standardized nature of these instruments reduces interviewer-induced variability. Nevertheless, residual information bias cannot be fully excluded and should be considered when interpreting the findings.

Furthermore, adequate prenatal care was operationalized solely based on the number of prenatal visits (six or more), following the minimum threshold recommended by Brazilian national guidelines. However, it is well recognized that the quality and content of prenatal care, including counseling, timely screening, and the therapeutic relationship with healthcare providers, are equally important determinants of treatment adherence and pregnancy outcomes. The absence of qualitative measures of prenatal care represents a limitation of this study. Additionally, clinical staging of syphilis was not systematically recorded, and adherence was defined based on the three-dose benzathine penicillin regimen. This approach may not reflect the appropriate treatment for women with primary or secondary syphilis, for whom a single dose is indicated, which represents a limitation of the present study. Also, sensitivity analyses considering maternal adherence alone were not performed, as 87% of women completed their own treatment, resulting in an insufficient number of non-adherent cases for reliable regression-based analyses. Finally, the cross-sectional design allows identification of associations, but not causal inferences.

### Statistical analysis

Statistical analyses were conducted using R Studio version 2024.09.1, adopting a significance level of p < 0.05. To identify factors associated with adherence to treatment, univariate binary logistic regressions were performed, estimating odds ratios (OR) and their 95% confidence intervals (CI). The proportion of participants with each factor was presented for both adherence and non-adherence groups. Variables significant at p < 0.05 in univariate analyses were subsequently entered into a multivariable logistic regression model using the enter method. Adjusted odds ratios (aOR) and 95% CIs are reported for the multivariable model. Multicollinearity was assessed by calculating the Variance Inflation Factor (VIF).

For the secondary outcomes (Apgar score and Capurro), groups with and without adherence were compared using independent-samples *t*-tests. These comparisons were further examined using analysis of covariance (ANCOVA), controlling for maternal age, income, and comorbidity status (hypertension or diabetes).

A co-occurrence network analysis was performed to examine relationships among binary factors significantly associated with syphilis treatment adherence during pregnancy. Using the igraph package in R, a binary dataset was constructed in which columns represented factors and rows represented individuals (0 = absence, 1 = presence). A co-occurrence matrix was generated by multiplying the transposed binary matrix by itself, so that each cell indicated the number of individuals presenting both factors. Community detection was then conducted with the Louvain algorithm to identify clusters of more densely connected nodes. Degree centrality was used to assess node importance, with higher values indicating greater co-occurrence and centrality in the network. Edges were weighted by co-occurrence frequency, and nodes and edges were color-coded according to cluster membership and strength of association. Separate networks were built for factors positively and negatively associated with treatment adherence in prior univariate analyses. Importantly, this was an exploratory analysis given that clusters indicate co-occurrence, not causality, and variable centrality does not imply etiological importance.

## Results

### Sample description

The sample had a mean age of 26.1 years (95% CI: 25.5–26.7) and an average monthly income of R$ 1586.90 (95% CI: R$ 1365.60–R$ 1808.10). A total of 27.3% of participants self-identified as Black (n = 89). Regarding the timing of syphilis diagnosis during pregnancy, the mean trimester of detection was 1.3 (95% CI: 1.2–1.4).

Concerning the primary outcome of treatment adherence, 43.6% (n = 142) of pregnant women and their partners completed full treatment for syphilis during the gestational period. Significant differences were observed in Apgar scores at the 1st minute (adherence – mean = 8.0 / SD = 1.3; non-adherence – mean = 7.5 / SD = 1.9; p = 0.009) and in gestational age estimated by the Capurro method (adherence – mean = 38.5 / SD = 1.5; non-adherence – mean = 37.8 / SD = 2.8; p = 0.005) between groups with and without adherence to the treatment. Additionally, the group effect of adherence remained significant for both outcomes after controlling for maternal age, income, and comorbidity status (hypertension or diabetes) (Apgar: p = 0.011; Capurro: p = 0.003). Notably, these were the most prevalent maternal comorbidities in the sample (hypertensive disorders: *n* = 93, 28%; diabetes: *n* = 58, 17%). Finally, it is worth noting that treatment adherence rates were considerably higher when only maternal treatment was considered (*n* = 293; 87%).

### Predictors of treatment adherence

A series of logistic regressions was conducted to identify factors significantly associated with adherence to syphilis treatment during pregnancy. Of the 42 candidate predictors, 18 were significantly associated with adherence (Table 1). Thirteen variables were negatively associated with adherence, including lifetime alcohol use disorder (OR: 0.31, 95% CI: 0.14–0.65, p = 0.003), lifetime cannabis use disorder (OR: 0.37, 95% CI: 0.18–0.73, p = 0.005), recent cannabis use (OR: 0.25, 95% CI: 0.08–0.64, p = 0.006), substance use during pregnancy (OR: 0.35, 95% CI: 0.14–0.75, p = 0.011), alcohol use during pregnancy (OR: 0.40, 95% CI: 0.18–0.83, p = 0.018), and a history of treatment for alcohol/drug problems (OR: 0.42, 95% CI: 0.19–0.87, p = 0.024). Additional factors linked to lower odds of adherence were any childhood maltreatment exposure (OR: 0.54, 95% CI: 0.34–0.84, p = 0.006), childhood physical neglect (OR: 0.60, 95% CI: 0.38–0.93, p = 0.023), lifetime physical violence victimization (OR: 0.47, 95% CI: 0.25–0.85, p = 0.014), lifetime witnessed violence (OR: 0.52, 95% CI: 0.31–0.86, p = 0.012), recent intimate partner conflict (OR: 0.47, 95% CI: 0.30–0.74, p = 0.001), recent unprotected sex (OR: 0.61, 95% CI: 0.38–0.98, p = 0.041), and a history of police detention (OR: 0.29, 95% CI: 0.14–0.57, p < 0.001).

**Table 1:** Logistic regression analysis of factors associated with adherence to gestational syphilis treatment during pregnancy.

**Factor**	**Adherence (N=142)**		**Non-adherence (N=183)**		**Odds ratio**	**95% CI Lower**	**95% CI Upper**	***P* value**
	**N**	**%**	**N**	**%**				
Adequate prenatal care	121	85.2	104	56.8	4.377	2.57	7.721	**0.000**
Early syphilis diagnosis	127	89.4	114	62.3	5.125	2.845	9.765	**0.000**
Prior police detention	11	7.7	41	22.4	0.291	0.137	0.571	**0.000**
Recent conflict with intimate partners	77	54.2	131	71.6	0.47	0.296	0.744	**0.001**
Planned pregnancy	40	28.2	26	14.2	2.368	1.37	4.155	**0.002**
Alcohol use disorder in life	9	6.3	33	18	0.311	0.135	0.648	**0.003**
Cannabis use disorder in life	12	8.5	36	19.7	0.374	0.18	0.731	**0.005**
Childhood maltreatment	71	50	119	65	0.538	0.343	0.84	**0.006**
Recent use of cannabis	5	3.5	23	12.6	0.254	0.084	0.635	**0.006**
Use of substance during pregnancy	8	5.6	27	14.8	0.345	0.142	0.752	**0.011**
Witnessed violence in life	28	19.7	59	32.2	0.516	0.305	0.859	**0.012**
Victim of physical violence	18	12.7	43	23.5	0.473	0.254	0.85	**0.014**
Employed	79	55.6	77	42.1	1.726	1.111	2.694	**0.015**
Income above Brazilian minimum wage	79	55.6	77	42.1	1.71	1.1	2.669	**0.017**
Use of Alcohol during pregnancy	10	7	29	15.8	0.402	0.18	0.83	**0.018**
Child physical neglect	65	45.8	107	58.5	0.6	0.384	0.932	**0.023**
Treatment for Alcohol/ Drug abuse in life	10	7	28	15.3	0.419	0.188	0.869	**0.024**
Recent unprotected sex in life	39	27.5	70	38.3	0.611	0.378	0.978	**0.041**
Tobacco use disorder in life	41	28.9	69	37.7	0.671	0.417	1.07	0.095
Recent use of alcohol	10	7	23	12.6	0.527	0.232	1.118	0.106
Receives government aid	76	53.5	82	44.8	1.418	0.914	2.206	0.119
Pregnancy history	78	54.9	116	63.4	0.704	0.45	1.1	0.123
Sexual abuse	3	2.1	10	5.5	0.373	0.083	1.248	0.140
History of Homelessness	7	4.9	17	9.3	0.506	0.191	1.211	0.142
Intervention of Child Protective Services (CPS)	3	2.1	1	0.5	3.928	0.497	79.877	0.238
Early sexual debut	58	40.8	86	47	0.779	0.499	1.212	0.268
Lack of recent social/familiar support	8	5.6	6	3.3	1.761	0.599	5.463	0.305
Child sexual abuse	11	7.7	20	10.9	0.684	0.307	1.455	0.334
Recent use of Tobacco	30	21.1	47	25.7	0.775	0.457	1.301	0.338
High school diploma	68	47.9	78	42.6	1.237	0.796	1.924	0.344
Common-law marriage	50	35.2	73	39.9	0.819	0.519	1.288	0.388
Age (> 21 gt; 21 years)	122	85.9	152	83.1	1.244	0.68	2.32	0.483
Black ethnicity	41	28.9	48	26.2	1.142	0.698	1.863	0.596
Psychiatric treatment in life	42	29.6	59	32.2	0.883	0.547	1.417	0.606
Child physical abuse	21	14.8	24	13.1	1.152	0.608	2.168	0.660
Depression/Anxiety diagnosis in life	45	31.7	54	29.5	1.108	0.688	1.782	0.671
Childhood emotional neglect	7	4.9	11	6	0.811	0.291	2.115	0.672
High-risk pregnancy	65	45.8	80	43.7	1.087	0.699	1.69	0.711
Tobacco use during pregnancy	39	27.5	53	29	0.929	0.568	1.51	0.766
Has > 1 close friend	109	76.8	141	77	0.966	0.571	1.645	0.898
Childhood emotional abuse	15	10.6	20	10.9	0.963	0.467	1.947	0.916
Resides in the capital city	108	76.1	140	76.5	0.976	0.584	1.64	0.925

**Note:** Data are presented as *n* (%) within each group. Odds ratios (ORs) and 95% confidence intervals (CIs) represent the odds of adherence (vs non-adherence) associated with each factor. *P* values were derived from Wald tests; statistical significance was defined as p < 0.05.

In contrast, five factors were positively associated with adherence: early diagnosis of syphilis (OR: 5.13, 95% CI: 2.85–9.77, p < 0.001), adequate prenatal care (OR: 4.38, 95% CI: 2.57–7.72, p < 0.001), planned pregnancy (OR: 2.37, 95% CI: 1.37–4.16, p = 0.002), current employment (OR: 1.73, 95% CI: 1.11–2.69, p = 0.015), and family income above the minimum wage (OR: 1.71, 95% CI: 1.10–2.67, p = 0.017).

### Co-occurrence network analysis

**Figure 1:**
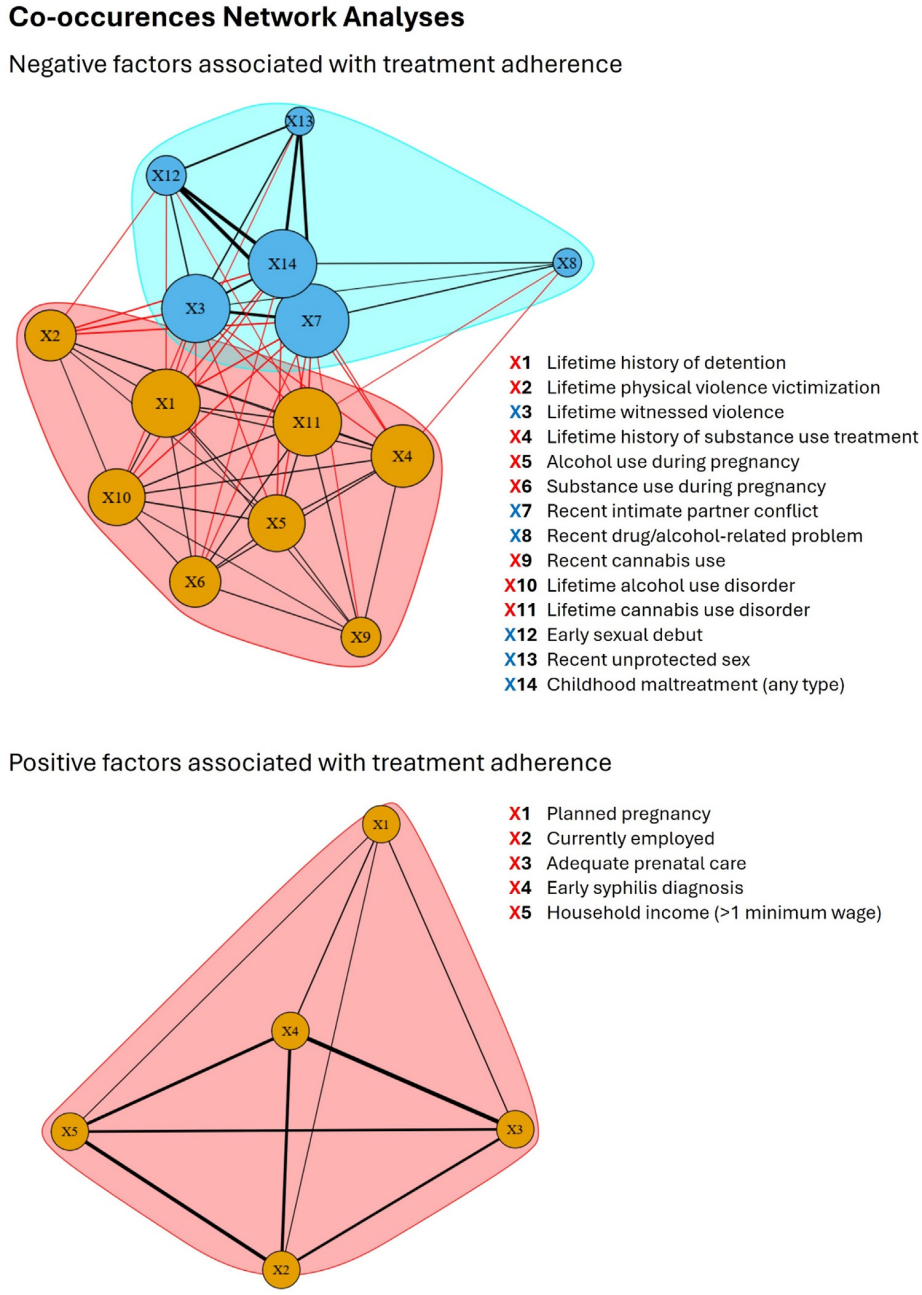
**Co-occurrence network analyses of factors associated with adherence to gestational syphilis treatment.** The upper panel displays variables negatively associated with treatment adherence, and the lower panel displays variables positively associated with adherence. Nodes represent the studied factors (coded as X1–X14 in the upper panel and X1–X5 in the lower panel, as listed in the figure). Edges represent pairwise co-occurrence relationships between factors; thicker lines indicate stronger relationships. Edge color denotes the direction of the association (black = positive; red = negative). Shaded areas and node colors indicate communities (clusters) of more strongly interconnected variables.

A co-occurrence network analysis was performed for the 13 variables negatively associated with treatment adherence. Community detection using the Louvain algorithm identified two main clusters: one related to psychosocial risks involving violence, intimate partner conflict, and sexual behavior (blue cluster, Figure 1), and another related to substance use patterns (red cluster).

The blue cluster included lifetime witnessed violence, recent intimate partner conflict, recent unprotected sex, and childhood maltreatment, including physical neglect. These variables were strongly interconnected, indicating a cohesive pattern of interpersonal violence and sexual risk behavior. The strongest co-occurrence in this cluster was between recent intimate partner conflict and childhood maltreatment.

The red cluster included police detention history, lifetime physical violence victimization, previous treatment for substance use, alcohol and substance use during pregnancy, recent cannabis use, and lifetime alcohol and cannabis use disorders. This cluster reflects a chronic vulnerability profile combining behavioral, legal, and violence-related risks. Bridging connections between the two clusters were also observed, particularly through recent intimate partner conflict, which showed the highest centrality in the network.

A separate co-occurrence network of the five factors positively associated with adherence formed a single cluster. Current employment, income above the minimum wage, adequate prenatal care, and early syphilis diagnosis were strongly interconnected, whereas planned pregnancy showed weaker links. These findings suggest that socioeconomic stability and access to prenatal care facilitate treatment adherence and may improve the management of syphilis during pregnancy.

### Multivariate regression analysis

In the multivariable logistic regression model including all previously identified variables significantly associated with treatment adherence, six variables remained independently associated with treatment adherence after mutual adjustment. Adequate prenatal care (aOR: 3.52, 95% CI: 1.80–6.89, p < 0.001) and early syphilis diagnosis (aOR: 2.94, 95% CI: 1.42–6.11, p = 0.004) remained the strongest positive predictors of adherence. Among negative predictors, lifetime alcohol use disorder (aOR: 0.07, 95% CI: 0.01–0.70, p = 0.023), history of police detention (aOR: 0.31, 95% CI: 0.11–0.89, p = 0.030), lifetime witnessed violence (aOR: 0.48, 95% CI: 0.25–0.94, p = 0.031), and recent intimate partner conflict (aOR: 0.48, 95% CI: 0.27–0.84, p = 0.011) were independently associated with lower odds of adherence. Adjusted odds ratios, 95% CIs, and p-values for all variables included in the multivariable model are reported in Table 2. Importantly, all VIF values were below 6, indicating no significant multicollinearity.

**Table 2:** Multivariable logistic regression analysis of factors associated with adherence to gestational syphilis treatment.

**Factor**	**aOR**	**95% CI**	**P-value**	**VIF**
***Positive predictors***				
Adequate prenatal care	3.52	1.80 – 6.89	**<0.001**	1.30
Early syphilis diagnosis	2.94	1.42 – 6.11	**0.004**	1.30
***Negative predictors***				
Prior police detention	0.31	0.11 – 0.89	**0.030**	1.81
Recent conflict with intimate partners	0.48	0.27 – 0.84	**0.011**	1.13
Alcohol use disorder in life	0.07	0.01 – 0.70	**0.023**	4.62
Witnessed violence in life	0.48	0.25 – 0.94	**0.031**	1.27
***Non-significant in adjusted model***				
Planned pregnancy	1.71	0.89 – 3.28	0.106	1.05
Cannabis use disorder in life	0.54	0.12 – 2.45	0.427	3.50
Childhood maltreatment	0.28	0.08 – 1.00	0.051	5.30
Recent use of cannabis	0.18	0.02 – 1.98	0.162	5.72
Use of substance during pregnancy	1.67	0.27 – 10.26	0.582	4.94
Victim of physical violence	2.75	0.84 – 9.06	0.095	1.33
Employed	0.85	0.28 – 2.60	0.780	4.61
Income above Brazilian minimum wage	1.46	0.49 – 4.36	0.498	4.54
Use of alcohol during pregnancy	7.94	1.00 – 62.70	0.050	4.47
Child physical neglect	1.83	0.51 – 6.54	0.352	5.32
Treatment for alcohol/drug abuse in life	7.82	0.75 – 81.28	0.085	5.37
Recent unprotected sex	0.63	0.35 – 1.12	0.116	1.08

**Note**: aOR, adjusted odds ratio; CI, confidence interval; VIF, variance inflation factor. Bold p-values indicate statistical significance (p < 0.05). All 18 variables significant in univariate analyses were entered simultaneously using the Enter method. VIF values were obtained from a linear regression model with identical predictors.

## Discussion

This study found that adherence to treatment for gestational syphilis is associated with socioeconomic, behavioral, and psychosocial factors. It was observed that only 43.6% had adequacy of treatment of the pregnant woman-partner dyad, a proportion similar to that reported in other Brazilian studies, which indicate adherence rates ranging from 35% to 55% [1,14]. Persistently low adherence remains one of the main barriers to eliminating vertical transmission of syphilis, a global goal set by the WHO [3].

Among the factors positively associated with adherence, adequate prenatal care, with six or more consultations, and early diagnosis of syphilis, during the first trimester of pregnancy, stood out. The variable "early diagnosis of syphilis" was present in approximately 89% of adherent pregnant women, compared to 62% of non-adherent ones. Timely initiation and quality of prenatal care, including early testing, are recognized as critical determinants of therapeutic adherence and prevention of congenital syphilis [15]. Early detection allows for immediate treatment, continuous counseling, and strengthening of the bond with the healthcare team, which promotes treatment compliance [16]. Pregnant women who begin follow-up early tend to receive diagnoses at initial stages and complete the treatment regimen before delivery [4]. Additionally, the participation of the sexual partner has also been crucial for therapeutic success, as partners should be evaluated and treated even when asymptomatic to prevent reinfection [17].

In socioeconomic terms, pregnancy planning, formal employment, and income above the minimum wage emerged as protective factors, reinforcing the impact of social determinants of health [18]. Job stability ensures greater financial autonomy, improved access to information, regular use of health services, and a better ability to adhere to medical appointments and treatments. These factors express the influence of social determinants on therapeutic behavior, as widely discussed in recent systematic reviews [19]. Labor market inclusion not only improves economic indicators but also serves as a social intervention capable of reducing health inequalities by expanding opportunities for access and use of health services and promoting greater adherence to treatment. Therefore, public policies aimed at job creation and social inclusion should be considered as complementary public health strategies.

On the other hand, psychosocial and behavioral factors were negatively associated with adherence, revealing contexts of multiple vulnerabilities. A history of incarceration, alcohol use, and illicit substance use is highly correlated with sexually transmitted infections, such as syphilis [20]. Supporting this notion, previous studies have demonstrated that substance use is associated with lower healthcare-seeking behavior and greater stigmatization, impeding prenatal follow-up and adherence to treatment [21]. Recent cannabis use and a history of treatment for alcohol or drug problems also emerged as risk factors, pointing to a chronic cycle of vulnerability and relapse, often associated with domestic violence and social exclusion.

In addition, the authors identified an association between experiences of violence and non-adherence to treatment. Recent conflict between intimate partners and lifelong exposure to violence emerged as central barriers, indicating a significant impact of gender-based violence and violent environments on treatment adherence. Evidence shows that exposure to physical, sexual, or emotional abuse reduces women’s autonomy, their ability to seek healthcare, and increases the risk of treatment interruption [22]. Such experiences weaken the bond with professionals, reduce trust in services, and generate anxiety and depressive symptoms that interfere with therapeutic continuity [23]. Furthermore, the results also highlighted the association between childhood trauma and adherence to treatment. Childhood abuse exposure has been shown to impair self-care behaviors and the ability to maintain trusting relationships with healthcare services [24]. This, in turn, might increase the likelihood of risk behaviors (e.g. early sexual initiation, unplanned pregnancy, substance use, etc.) and lower therapeutic adherence [[25], [26], [27], [28]].

The co-occurrence network analysis provided a complementary perspective by identifying two exploratory and distinct clusters associated with vulnerability profiles related to non-adherence to treatment. The blue cluster, encompassing childhood maltreatment, intimate partner conflict, witnessed violence, and recent unprotected sex, reflects a pattern consistent with the theory of polyvictimization, where exposure to one form of violence substantially increases the likelihood of exposure to others across the life course [29]. This clustering suggests that, for a subset of women, non-adherence to syphilis treatment may be embedded within a broader context of interpersonal violence and relational instability that extends beyond the healthcare encounter. The red cluster, comprising substance use disorders, police detention, physical violence victimization, and prior treatment for alcohol and drug problems, reflects a chronic vulnerability profile in which legal marginalization, addiction, and violence intersect. These overlapping adversities may constrain women’s capacity to engage consistently with prenatal care and to complete syphilis treatment. In contrast, the single cluster formed by positive predictors highlights the role of socioeconomic stability and healthcare access as mutually reinforcing protective factors.

However, it should be noted that after adjustments in the multivariable model, only six variables remained independently associated with treatment adherence. Adequate prenatal care and early syphilis diagnosis remained the strongest positive predictors of adherence, while among negative predictors, lifetime alcohol use disorder showed the strongest independent association with non-adherence. History of police detention, lifetime witnessed violence, and recent intimate partner conflict also remained independently associated with non-adherence, underscoring that legal vulnerability, exposure to violence, and substance misuse potentially operate as distinct barriers for gestational syphilis treatment adherence.

Regarding neonatal outcomes, significant differences were observed between groups. Specifically, Apgar scores at the first minute and gestational age assessed by the Capurro method suggest that adherence to gestational syphilis treatment has an important impact on newborn vitality and maturity at birth. Although these findings should be interpreted with caution due to potential social and obstetric confounding, they remained significant after controlling for maternal age, income, and comorbidity status related to hypertensive disorders and diabetes. Infants born to adherent mothers had higher first-minute Apgar scores and greater gestational age by the Capurro method, indicating a lower likelihood of prematurity and fetal compromise. These findings are consistent with previous studies showing that adequate syphilis treatment during pregnancy is associated with fewer adverse outcomes, including preterm birth, low birth weight, and neonatal asphyxia [30]. Thus, adequate and timely treatment stands as one of the most effective interventions to reduce neonatal morbidity and mortality related to syphilis [31].

Beyond the limitations of the cross-sectional design, which warrant cautious interpretation, these findings support the concept of “complex vulnerability,” in which individual and socioeconomic factors are associated with impaired therapeutic adherence. Addressing this scenario requires coordinated public policies that strengthen prenatal care, expand harm reduction and psychosocial support, and confront violence and substance use within reproductive health programs, alongside intersectoral actions to promote women’s empowerment and health equity.

## Funding sources

This study received no funding.

## Data availability

The data that support the findings of this study are available from the corresponding author upon reasonable request.

## Conflicts of interest

The authors declare no conflicts of interest.
